# Cyclophosphamide abrogates the expansion of CD4**^+^**Foxp3**^+^** regulatory T cells and enhances the efficacy of bleomycin in the treatment of mouse B16-F10 melanomas

**DOI:** 10.20892/j.issn.2095-3941.2021.0027

**Published:** 2021-08-11

**Authors:** Ping Li, Fengyang Chen, Jingbin Zheng, Yang Yang, Yuan Li, Yifei Wang, Xin Chen

**Affiliations:** 1 State Key Laboratory of Quality Research in Chinese Medicine, Institute of Chinese Medical Science, University of Macau, Macau 999078, China

**Keywords:** Bleomycin, cyclophosphamide, tumor necrosis factor, Tregs, TNFR2

## Abstract

**Objective:**

Promotion of the proliferative expansion of CD4^+^Foxp3^+^ regulatory T cells (Tregs) is one of the side effects that limits the use of bleomycin (BLM) in the treatment of tumors. In this study, we examined the hypothesis that cyclophosphamide (CY), a chemotherapeutic agent with the capacity to eliminate tumor infiltrating Tregs, abrogated BLM-induced expansion of Tregs and consequently resulted in a better anti-tumor effect.

**Methods:**

The *in vitro* effects of BLM, with or without mafosfamide (MAF, the active metabolite of CY), on both TGF-β-induced differentiation of Tregs (iTregs), and TNF-induced expansion of naturally occurring Tregs (nTregs) were assessed. The *in vivo* effect of low doses of BLM and CY on tumor-infiltrating Tregs, as well as on the growth of mouse B16-F10 melanomas, was also studied.

**Results:**

*In vitro* treatment with BLM promoted the differentiation of iTregs, as well as TNF-induced expansion of nTregs. These effects of BLM were completely abrogated by MAF. Furthermore, in the mouse B16-F10 melanoma model, treatment with low doses of BLM increased the number of tumor-infiltrating Tregs, and this effect of BLM was also abrogated by CY. Importantly, combination therapy with low doses of BLM and CY showed synergistic anti-tumor effects.

**Conclusions:**

CY abrogated the effect of BLM on the expansion of Tregs. The combination of these 2 chemotherapeutic agents may represent a safer and more effective therapy in the treatment of cancer patients, and thus merits future clinical evaluation.

## Introduction

Bleomycin (BLM) is a glycopeptide antibiotic derived from the bacterium, Streptomyces^1^. BLM is used in the treatment of different types of cancer, including skin carcinoma, lymphoma, ovarian cancer, and non-small cell lung cancer^1,2^. BLM lacks bone marrow toxicity^3^, but can induce idiopathic pulmonary fibrosis (IPF) in patients, which hampers its clinical use^4,5^. One strategy to mitigate its toxic effect is to use low doses of BLM in conjunction with other chemotherapeutic agents^6,7^. This combination therapy is able to preserve the anti-tumor effect of BLM while only causing minor and reversible lung injury^8^.

Another major adverse effect of BLM treatment is its capacity to induce expansion of CD4^+^Foxp3^+^ regulatory T cells (Tregs), presumably by stimulating the production of TGF-β^9,10^. There is compelling evidence that Tregs represent a major immunosuppressive mechanism in cancer immune evasion^11,12^. Thus, expansion of Tregs can offset the anti-tumor effect of BLM^9,13^. Indeed, it was shown that depletion of Tregs enhanced the anti-tumor effect of BLM^9^. Furthermore, expansion of Tregs exacerbated BLM-induced pulmonary fibrosis^14^. Therefore, addition of a therapeutic agent with the capacity to eliminate Tregs may further enhance the anti-tumor effects of BLM while reducing lung injury induced by BLM. Cyclophosphamide (CY) has the capacity to selectively reduce the number of highly immunosuppressive tumor necrosis factor receptor 2 (TNFR2)-expressing Tregs in the tumor microenvironments^15^. We therefore determined if CY abrogated the expansion of Tregs induced by treatment with BLM, to consequently enhance its anti-tumor effect.

In this study, we report for the first time that BLM promoted the differentiation of TGF-β-induced Tregs (iTregs) and stimulated the expansion of naturally occurring Tregs (nTergs) induced by TNF *in vitro*. This effect of BLM was abrogated by mafosfamide (MAF), the active metabolite of CY^16^. We confirmed that *in vivo* treatment with BLM at a relatively low dose induced the systemic expansion of Tregs in mouse malignant tumor models, including B16-F10 melanomas. This effect of BLM was abrogated by treatment with CY. Importantly, a combination of BLM and CY resulted in a synergistic anti-tumor effect. The results provided evidence that a combination of low doses of BLM with CY may be a novel therapeutic approach in the treatment of cancer patients.

## Materials and methods

### Mice, cells, and reagents

Female C57BL/6 mice (8–12 weeks of age) were obtained from the Jackson Laboratory (Bar Harbor, ME, USA), and were maintained under specific pathogen-free conditions in the Animal Facility of the University of Macau. The animal research protocol was approved by the Animal Research Ethics Committee of the University of Macau.

Both mouse Lewis lung carcinoma (LLC) and mouse melanoma B16-F10 cell lines were purchased from the American Type Culture Collection (Manassas, VA, USA). The LLC and B16-F10 cell lines were cultured in *Dulbecco’s Modified Eagle Medium* supplemented with 10% fetal calf serum, 100 units/mL penicillin, and 100 µg/mL streptomycin at 37 °C and 5% CO_2_ in a humidified incubator.

Bleomycin sulfate was purchased from Ark Pharm (Arlington Heights, IL, USA). Cyclophosphamide monohydrate was purchased from Sigma-Aldrich (St. Louis, MO, USA). Mafosfamide sodium salt was purchased from Santa Cruz Biotechnology (Santa Cruz, CA, USA). Antibodies including PerCP-Cy5.5 anti-mouse TCRβ (H57-597), PerCP-Cy5.5 anti-mouse CD4 (RM4-5), and PE anti-mouse CD120b (TNFR2, TR75-89) were purchased from BD Pharmingen (San Diego, CA, USA). Antibodies termed PE-Cy7 anti-mouse CD4 (GK1.5), eFluor 450 anti-CD4 (RM4-5), PerCP-Cy5.5 anti-mouse CD45 (30-F11), APC anti-mouse/rat Foxp3 (FJK-16s), mouse TGF beta 1 Recombinant Protein (rmTGF-β), and Foxp3/Transcription Factor Staining Buffer Set were purchased from Invitrogen (Carlsbad, CA, USA). Anti-CD3, anti-CD28, anti-Recombinant Mouse TNF (rmTNF), and rmIL-2 were purchased from BD Pharmingen (San Jose, CA, USA). The LIVE/DEAD Fixable Near-IR Dead Cell Stain Kit and CellTrace™ Violet were purchased from Thermo Fisher Scientific (Waltham, MA, USA).

### *In vitro* generation and detection of Treg cells

CD4^+^ T cells were isolated from lymphocytes of C57BL/6 mice using Mouse CD4 (L3T4) MicroBeads and MS Columns (Miltenyi Biotec, Bergisch Gladbach, Germany). The purity of the isolated cells was > 95%. In a U-shaped 96-well plate, cells (1 × 10^5^ cells/well) were subsequently activated by plate-coated anti-CD3e antibody (1 µg/mL) and soluble anti-CD28 antibody (0.5 µg/mL), with rmTGF-β (5 ng/mL). BLM or MAF was added into wells as indicated. After culturing for 2 days, the cells were collected, fixed, and permeabilized. Foxp3 expression by CD4^+^ cells was determined by flow cytometry.

### Conditions for TNF-induced nTreg proliferation *in vitro*

In a U-shaped 96-well plate, CellTrace™ Violet-labeled CD4^+^ T cells (1 × 10^6^ cells/well) were stimulated with rmIL-2 (0.25 ng/mL) plus rmTNF (5 ng/mL), in the presence or absence of BLM or MAF. After culturing for 3 days, the cells were collected, fixed, and permeabilized. The replicating Foxp3^+^ CD4^+^ T cells and the expression of TNFR2 were analyzed by flow cytometry.

### Tumor cell inoculation and treatment

LLC or B16-F10 melanoma cells [500,000 cells in 0.1 mL phosphate-buffered saline (PBS)] were injected subcutaneously into the right flank of C57BL/6 mice. Tumor size was calculated using the formula: (length × width^2^)/2. Treatment was started on day 6 after tumor inoculation. LLC tumor-bearing mice were treated intravenously (i.v.) with BLM (2 mg/kg in PBS) every 3 days for a total of 4 times. B16-F10 melanoma-bearing mice were treated with BLM (2 mg/kg in PBS, i.v.) every other day for a total of 4 times. For BLM and CY combination therapy, BLM (2 mg/kg, i.v.) was administered every 3 days for 4 times, starting from day 6 after inoculation of B16-F10 melanoma cells, and CY (50 mg/kg, in PBS) was administrated every 3 days by intraperitoneal injection for 4 doses starting on day 7 after inoculation. On day 17 after tumor inoculation, the mice were euthanized and their spleens, lymph nodes, and tumor tissues were harvested for fluorescence-activated cell sorting (FACS) analysis and identification of Tregs.

### Characterization of tumor infiltrating lymphocytes

At the indicated times, tumors were excised, minced, and digested with RPMI 1640 containing 0.1 mg/mL DNase I, and 1 mg/mL collagenase IV. The fragments were pushed through a 70 µm pore size cell strainer to obtain a single cell suspension. Cells were stained with the LIVE/DEAD Fixable Near-IR Dead Cell Stain Kit, then fixed and permeabilized. The percentages of Foxp3^+^ cells gating on live CD45^+^ CD4^+^ T cells and exhibiting TNFR2 expression were determined by flow cytometry.

### Flow cytometry

After blocking Fc receptors, the cells were incubated with appropriately diluted antibodies and finally suspended in fluorescence-activated cell sorting (FACS) buffer for cytometric analysis. Acquisition was performed using BD LSRFortessa flow cytometry (BD Biosciences, San Jose, CA, USA). FlowJo software (Tree Star, Ashland, OR, USA) was used for data analysis. The gating strategy for tumor infiltrating Tregs (CD4^+^Foxp3^+^) is shown in **Supplementary Figure S1**.

### Hematoxylin and eosin (H&E) staining

Mouse lungs were harvested and washed in PBS to remove blood. The tissues were then fixed in 4% paraformaldehyde for 24 h at 4 °C. Lung tissues were first dehydrated in gradient alcohol solutions and then embedded in paraffin. Four micrometre sections were then cut and collected for H&E staining.

### Statistical analysis

All data are presented as means ± SD. The statistical analysis was performed using a *t-*test or one-way analysis of variance using Prism 7.0 software (GraphPad, San Diego, CA, USA). A *P* value < 0.05 was considered to be statistically significant.

## Results

### The effects of low dose of BLM on the number of infiltrating Tregs in mouse tumor models

**Figure 1:**
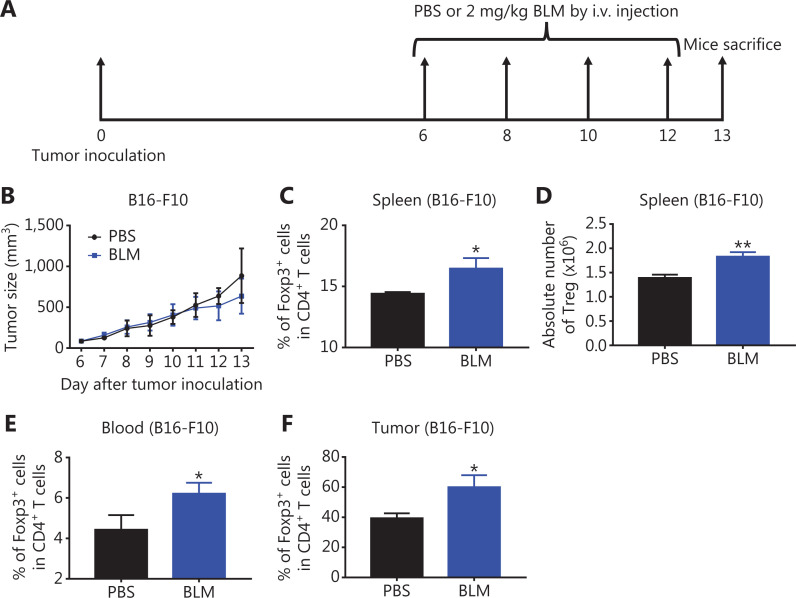
Treatment with low doses of bleomycin (BLM) increased the number of regulatory T cells (Tregs) in B16-F10 melanoma-bearing mice. C57BL/6 mice were inoculated in the right flank with B16-F10 melanoma cells [500,000 cells in 0.1 mL of phosphate-buffered saline (PBS)]. On day 6 after inoculation, mice were treated with PBS, or 2 mg/kg BLM every other day for a total of 4 doses. On day 13 after tumor inoculation, mice were sacrificed. The percentages of Tregs in CD4^+^ T cells in the spleen, blood, and tumor were analyzed by fluorescence-activated cell sorting. (A) Schematic of the experimental protocol. (B) The growth curves of B16-F10 melanomas in mice. (C, E, F) The percentages of Tregs in CD4^+^ T cells in the spleen, blood, and tumor tissue. (D) The absolute number of Tregs in the spleen. Data (means ± SD, *N* = 5) shown are representatives of 2 separate experiments with similar results. *P < 0.05 and **P < 0.01 *vs.* the PBS group.

It was reported that treatment with BLM at 20 mg/kg resulted in the expansion of Tregs in mice bearing CT26 colon cancers^9^, and that this dose of BLM was toxic and could cause lung injuries^17^. To determine the effect of BLM on Tregs using a nontoxic dose to the lungs, B16-F10 melanoma-bearing mice were injected i.v. with 2 mg/kg of BLM every other day for 4 times, starting from day 6 after tumor cell inoculation (as shown in **Figure 1A**). We confirmed that BLM with this low dose did not cause lung injuries (**Supplementary Figure S2**), and that this treatment did not result in inhibition of B16-F10 melanoma tumor growth (**Figure 1B**, *P* > 0.05). However, the percentage of Foxp3^+^ cells in CD4^+^ T cells was increased in the spleen, blood, and tumor tissue by 15%–50% in mice treated with BLM (**Figure 1C, E–F**, *P* < 0.05). The absolute number of Tregs in the spleen was also increased by BLM treatment (**Figure 1D**, *P* < 0.01). Similar observations were made in mice bearing LLC Lewis lung cancers, although treatment with BLM resulted in increased inhibition of tumor growth (*P* < 0.05, **Supplementary Figure S3**). Together, the results showed for the first time that, even at a lower dose, treatment with BLM increased the number of Tregs in tumor bearing mice.

### BLM promoted the *in vitro* differentiation of iTregs, which was inhibited by MAF

**Figure 2:**
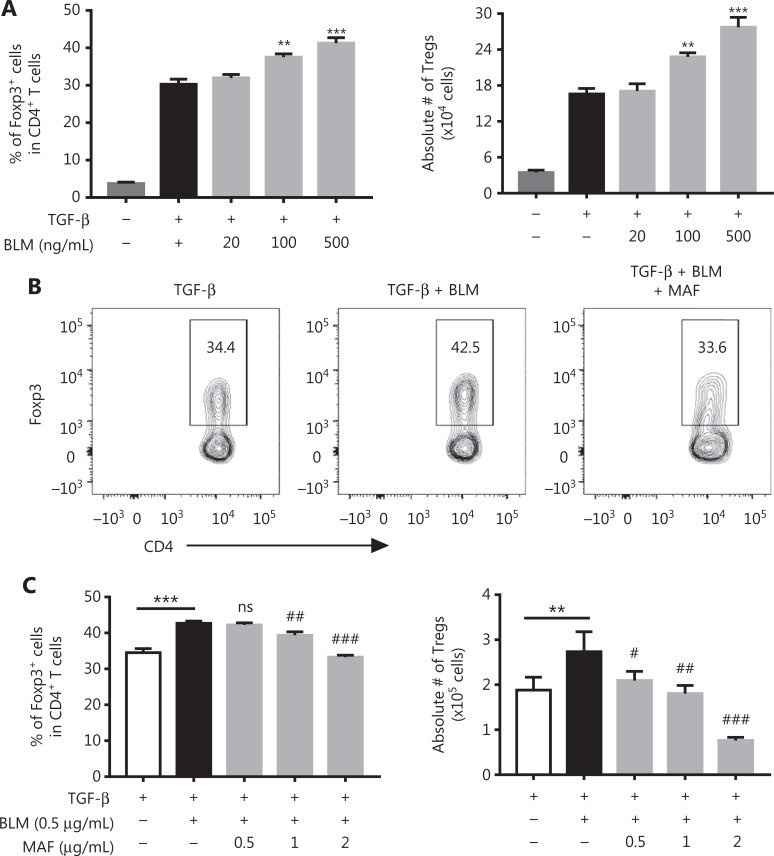
The effects of bleomycin (BLM) and mafosfamide (MAF) on the differentiation of TGF-β-induced differentiation of Tregs. Purified CD4^+^ T cells were stimulated with anti-CD3/CD28 antibodies in the presence of TGF-β. The cells were then treated with the indicated concentrations of BLM with or without MAF. After 48 h, the percentage of Foxp3^+^ cells was analyzed by fluorescence-activated cell sorting (FACS) and the absolute number of Foxp3^+^ cells was determined. (A) BLM promoted TGF-β-induced iTreg differentiation. (B) Typical FACS plots. (C) MAF inhibited the effect of BLM on the promotion of iTreg differentiation. Data (means ± SD, *N* = 3) shown are representatives of 3 separate experiments with similar results. **P < 0.01; ***P < 0.001 *vs*. the TGF-β treatment group. ^#^*P* < 0.05; ^##^*P* < 0.01; ^###^*P* < 0.001 *vs.* the BLM treatment only group.

Increases in Tregs could have resulted from either the conversion of naïve CD4^+^ T cells into iTregs or from the proliferative expansion of pre-existing nTregs^18–20^. To clarify the mechanism underlying the effect of BLM on the expansion of Tregs, we examined the effect of BLM on the differentiation of iTregs. To this end, purified CD4^+^ T cells were activated with anti-CD3 and anti-CD28 antibodies, in the presence of TGF-β, for 48 h, and the number and the percentage of Foxp3-expressing Tregs were determined. **Figure 2A** shows that TGF-β-induced Foxp3 expression in CD4^+^ T cells was enhanced by BLM over concentration ranges of 100–500 ng/mL (*P* < 0.001–0.01). The absolute number of Tregs was also increased by BLM treatment (*P* < 0.001–0.01).

MAF is a derivative of CY, which is active without being further metabolized. So it can be used for *in vitro* studies^16^. We found that MAF (0.5–2 µg/mL) inhibited BLM-induced increases in Tregs, including both the percentage of Foxp3^+^ cells in CD4^+^ T cells (**Figure 2B and 2C**, *P* < 0.001–0.01) and the absolute number of Tregs (**Figure 2C**, *P* < 0.001–0.05). Thus, MAF inhibited BLM-induced differentiation of iTregs.

### BLM augmented TNF-induced proliferative expansion of nTregs *in vitro*, which was also inhibited by MAF

**Figure 3:**
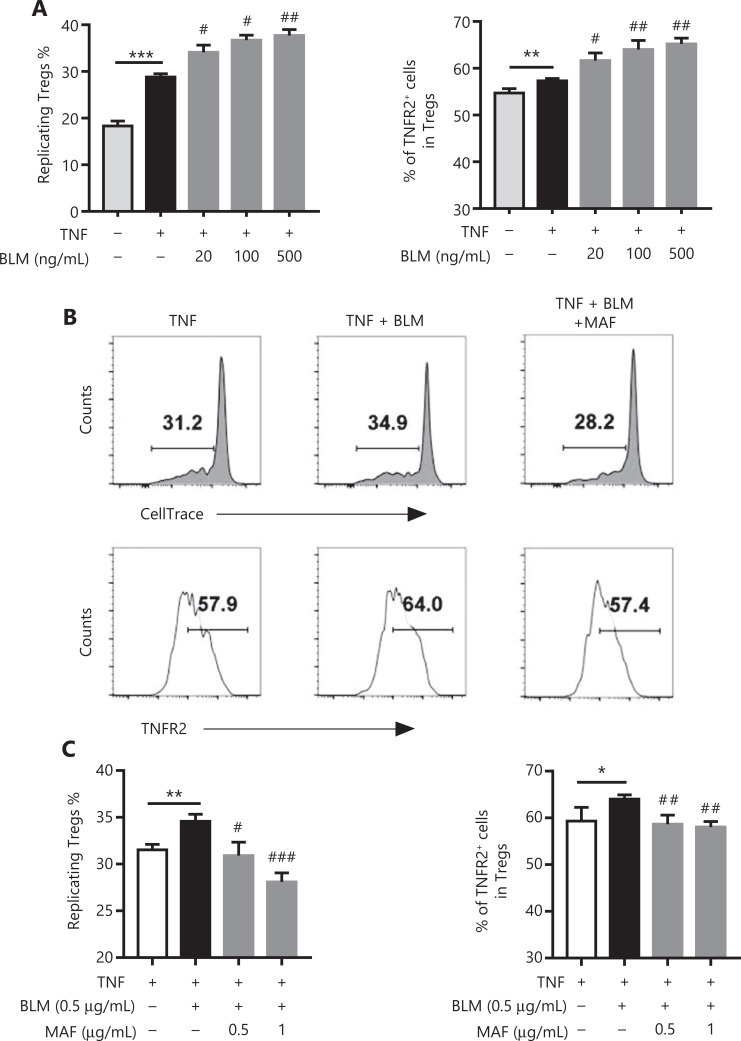
The effect of bleomycin (BLM) and mafosfamide (MAF) on the expansion and TNFR2 expression of naturally occurring regulatory T cells (Tregs) induced by TNF. Purified CD4^+^ cells were labeled with CellTrace™ Violet and cultured in medium containing IL-2 in the presence or absence of TNF. The cells were treated with the indicated concentrations of BLM with or without MAF. After 3 days, the percentage of replicating Tregs, based on the dilution of the CellTrace™ Violet signal, and the percentage of TNFR2^+^ Tregs were analyzed by fluorescence-activated cell sorting (FACS), by gating of CD4^+^Foxp3^+^ cells. (A) The effect of BLM on TNF-induced naturally occurring Treg (nTreg) proliferation and TNFR2 expression. Data are the summary of results pooled from 3 independent experiments. (B) Typical FACS plots and (C) MAF inhibited the effect of BLM on the promotion of nTreg expansion induced by TNF. Data (means ± SD, *N* = 3) shown are representatives of 3 separate experiments with similar results. *P < 0.05; **P < 0.01; ***P < 0.001 *vs.* the TNF treatment group. ^#^*P* < 0.05; ^##^*P* < 0.01; ^###^*P* < 0.001 *vs.* the BLM treatment only group.

By stimulating TNFR2, we previously reported that *in vitro* stimulation with TNF resulted in the preferential proliferation of pre-existing nTregs present in CD4^+^ T cells^21–23^. We therefore determined the effect of BLM on nTreg expansion in this experimental setting. To this end, purified CD4^+^ T cells were cultured in IL-2-containing medium (0.25 ng/mL) to maintain the survival of Tregs. The proliferation of Tregs was assessed by gating of Foxp3^+^ cells, based on the dilution of CellTrace™ Violet. **Figure 3A** shows that the addition of TNF (5 ng/mL) resulted in a 44% increase in proliferation of Tregs (*P* < 0.001). Notably, the proliferation of Tregs and TNFR2 expressions of Tregs induced by TNF was further increased by treatment with BLM (**Figure 3A**, *P* < 0.01–0.05). This effect of BLM was inhibited by MAF at concentrations starting from 0.5–1 µg/mL (*P* < 0.001–0.05, **Figure 3B and 3C**), showing that BLM also promoted the proliferation of nTregs, which was abrogated by treatment with MAF.

### CY abrogated the proliferation of Tregs induced by BLM treatment in B16-F10 melanoma-bearing mice

**Figure 4:**
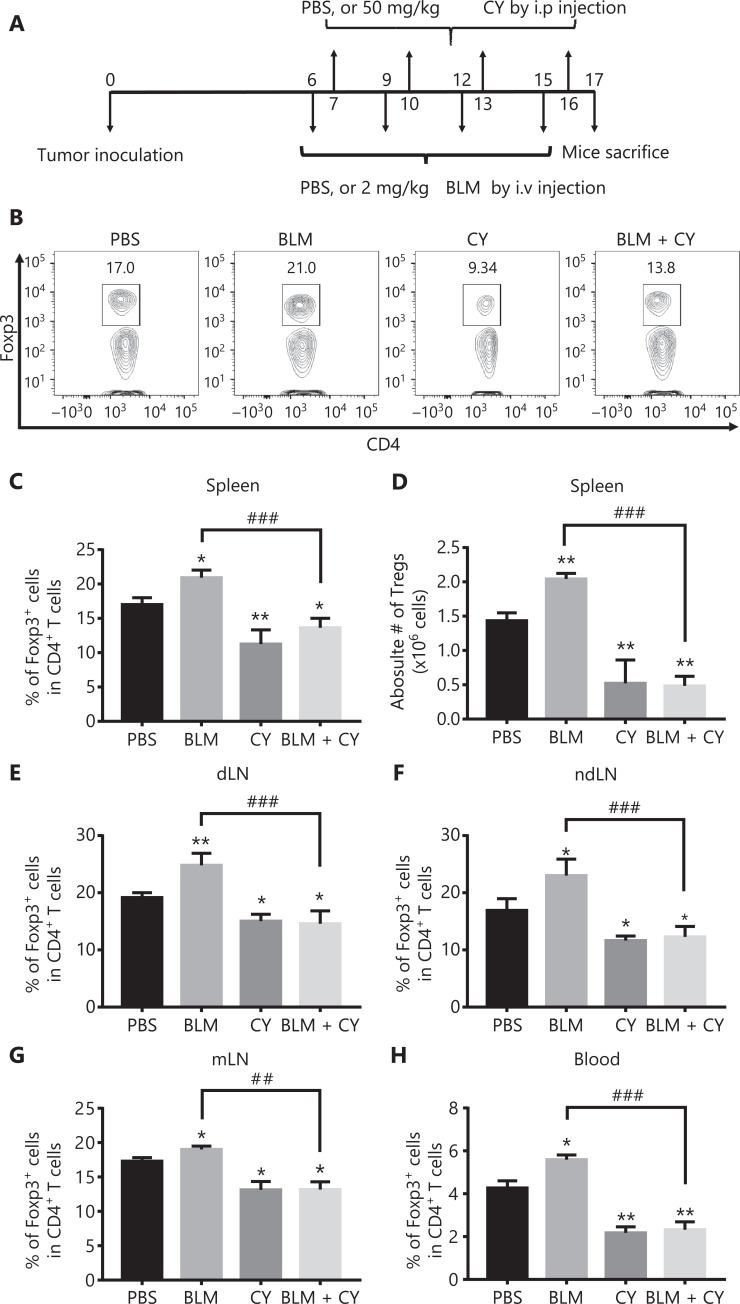
CY inhibited bleomycin (BLM)-induced expansion of regulatory T cells (Tregs) in the spleen and lymph nodes (LNs) of B16-F10 melanoma-bearing mice. C57BL/6 mice were inoculated in the right flank with B16-F10 melanomas [500,000 cells in 0.1 mL of phosphate-buffered saline (PBS)]. On day 6 after tumor inoculation, mice were treated with PBS, BLM, CY, or BLM plus CY. At day 17 after B16-F10 melanoma inoculation, mice were sacrificed. The percentage of Foxp3-expressing Tregs in CD4^+^ T cells present in the spleen and LNs was analyzed by fluorescent-activated cell sorting. (A) Schematic of the experimental protocol. (B, C) Percentage of Foxp3^+^ cells gating on CD4^+^ T cells in the spleen. (B) Typical flow plots. (C) Summary of the percentage of Foxp3^+^ cells in CD4^+^ T cells in the spleen, LNs, and blood (means ± SD, *N* = 5). (D) Summary of the absolute number of Tregs in the spleen (means ± SD, *N* = 5). (E–H) Summary data of the percentage of Foxp3^+^ cells in CD4^+^ T cells in draining lymph nodes, non-draining lymph nodes, mesenteric lymph nodes, and blood (means ± SD, *N* = 5). *P < 0.05; **P < 0.01 *vs.* the PBS treatment group; ^##^*P* < 0.01; ^###^*P* < 0.001 *vs.* the indicated groups. Data shown are representative of 2 separate experiments with similar results.

We next determined if CY abrogated the *in vivo* effect of BLM on Treg expansion and consequently enhanced its anti-tumor effect. To this end, B16-F10 melanoma-bearing mice were injected i.v. with BLM (2 mg/kg) every 3 days starting from day 6 after tumor inoculation, for a total of 4 doses. The mice were also injected i.p. with CY (50 mg/kg) for 4 doses, every 3 days starting from day 7 after tumor inoculation (**Figure 4A**). On day 17 after tumor inoculation, the mice were sacrificed and the percentages of Tregs in the spleen, draining lymph nodes (dLNs), non-draining LNs (ndLNs), mesenteric LNs (mLNs), and tumor tissues were analyzed by flow cytometry. **Figure 4** shows that monotherapy with CY decreased the percentages of Tregs in lymphoid tissues and the blood (*P* < 0.01–0.05), as compared with PBS treatment. Importantly, increases in the percentages of Tregs in the spleen (**4B–C**), dLN (**4E**), ndLN (**4F**), mLN (**4G**), and blood (**4H**) were completely abrogated by treatment with CY (**Figure 4**, *P* < 0.001–0.01). Treatment with CY also reduced the number of Tregs in the spleen in BLM-treated mice (**Figure 4D**, *P* < 0.001).

**Figure 5:**
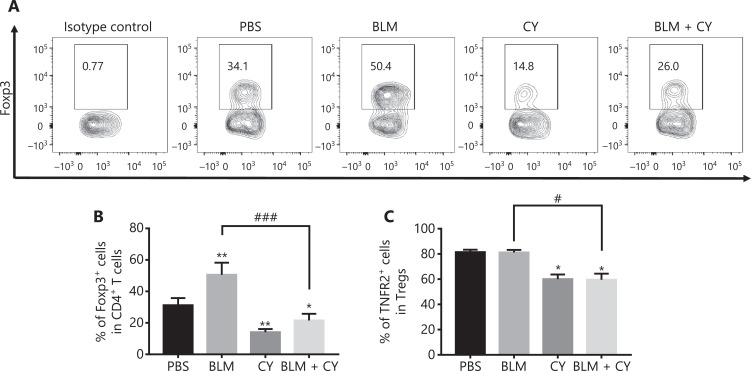
The effect of cyclophosphamide on bleomycin-induced expansion of regulatory T cells (Tregs) in tumor tissue. The B16-F10 melanoma model and its treatment are described in **Figure 4A**. The percentage of Foxp3-expressing Tregs and TNFR2 expression on Tregs in tumor infiltrating CD45^+^CD4^+^ cells was analyzed by fluorescence-activated cell sorting. (A, B) The percentage of Foxp3^+^ cells present in live CD45^+^ CD4^+^ cells infiltrating tumor tissues. (A) Typical flow plots. (B) Summary of the percentage of Foxp3^+^ Tregs and (C) percentage of TNFR2^+^ Tregs. Data (means ± SD, *N* = 5) shown are representatives of 2 separate experiments with similar results. *P < 0.05, **P < 0.01 *vs*. the phosphate-buffered saline treatment group; ^#^*P* < 0.05; ^###^*P* < 0.001 *vs.* the indicated groups.

The percentage of Tregs in tumor infiltrating CD4^+^ T cells was 34.1%, which was increased by treatment with BLM to 50.4% and decreased by treatment with CY to 14.8% (**Figure 5**). Importantly, the increase of Tregs in BLM-treated mice was completely abrogated by CY treatment (down to 26%, *P* < 0.001) (**Figure 5A, B**). We have previously reported that TNFR2 was associated with a highly suppressive phenotype of Tregs, and that this marker was characteristically expressed by the majority of tumor-infiltrating Tregs^22,24^. Consistent with a previous report^15^, CY treatment, alone or with BLM, also reduced the number of TNFR2-expressing Tregs in tumor tissues (**Figure 5C**) (*P* < 0.05), suggesting that CY reduced the number of tumor infiltrating Tregs and also decreased their suppressive capacity and proliferative expansion.

### Combination therapy with CY and BLM synergistically inhibited the growth of B16-F10 melanomas in mice

**Figure 6:**
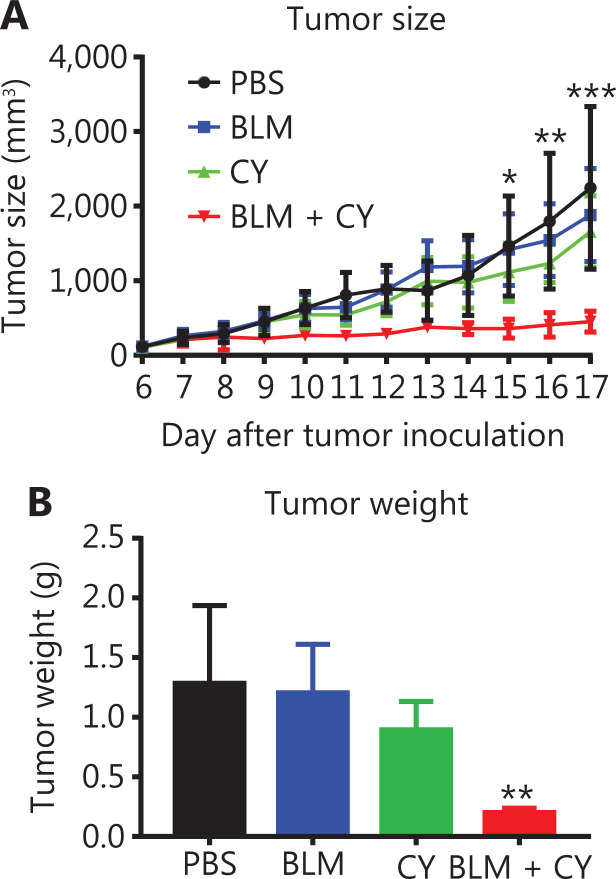
The effect of cyclophosphamide and bleomycin on the inhibition of growth of B16-F10 melanomas. The B16-F10 melanoma model and its treatment are described in **Figure 4A**. (A) The growth curves of B16-F10 melanomas in mice. (B) The weight of tumors at 17 days after B16-F10 melanoma inoculation. The data (means ± SD, *N* = 5) shown are representatives of 2 separate experiments with similar results. *P < 0.05; **P < 0.01; ***P < 0.001 *vs.* the PBS treatment group.

We further determined the effect of combination therapy of BLM and CY on the growth of B16-F10 melanomas in syngeneic C57BL/6 mice. **Figure 6** shows that monotherapy with CY or BLM only resulted in a modest inhibition of B16-F10 growth of melanomas in mice (*P* > 0.05). However, the combination of BLM and CY resulted in an enhanced inhibition B16-F10 melanoma growth (**Figure 6A**) (*P* < 0.001–0.05). Compared with the PBS-treated group, the tumor weight was decreased 80% after combination therapy (**Figure 6B**) (*P* < 0.01). In addition, we confirmed that BLM alone at doses used in this study or in combination with CY did not cause lung injuries (**Supplementary Figure S4**). Together, the results showed that BLM and CY showed excellent efficacy for the growth inhibition of mouse B16-F10 melanomas.

## Discussion

In this study, we clearly showed that CY was able to completely eliminate the stimulatory effect of BLM on Tregs, and consequently improve its anti-tumor efficacy. IPF is a major complication of BLM treatment in cancer patients^25,26^. In addition to decreasing anti-tumor immune responses, expansion of Tregs induced by BLM may cause the development of IPF^14^. Thus, CY may also ameliorate BLM-induced IPF through elimination of Tregs. To mimic the clinical application, our study used a relatively low dosage of BLM that did not cause lung injuries. However, further investigation is required to completely evaluate the effects of CY on BLM-induced IPF in an appropriate animal model.

It was previously reported that BLM-induced increases in Tregs were associated with upregulation of TGF-β mRNA expression^9^. In this study, we determined the protein levels of TGF-β in the serum of melanoma-bearing mice, but failed to find any difference in response to BLM treatment (data not shown). This may be because we used a lower dosage of BLM in our study, as compared with that in a previous report^9^. Furthermore, induction of TGF-β production by BLM is a time-dependent event^27^, and we may not have measured this cytokine at an optimal time frame. In addition, serum may not reflect the tissue levels of TGF-β. Nevertheless, we found that the level of TNF in the serum of BLM-treated melanoma-bearing mice was increased (data not shown). This result is consistent with previous reports showing that high levels of TNF were induced by BLM in a mouse lung injury model^10,28^. To identify the mechanism underlying the stimulatory effect of BLM on Treg expansion, we examined the effect of BLM on the differentiation of iTregs induced by TGF-β and on the expansion of nTergs induced by TNF *in vitro*. To this end, unfractionated CD4^+^ T cells were used because, similar to naïve CD4^+^ T cells, their Foxp3 expression can be induced and upregulated by TGF-β^29^. Furthermore, the number of Foxp3-expressing Tregs in CD4^+^ T cells can be expanded by TNF stimulation in a similar manner as in purified CD4^+^Foxp3^+^ Tregs^21^. The results of our study for the first time showed that BLM had the capacity to promote *in vitro* iTreg differentiation and nTreg expansion. Because BLM treatment was able to increase TNF levels in mouse serum (data not shown) and CY, an agent known to eliminate TNFR2-expressing Tregs^15^, was able to abrogate this expansion of Tregs in BLM-treated mice, we hypothesized that the enhanced interaction of TNF-TNFR2 was responsible for the expansion of Tregs by *in vivo* BLM treatment. Nevertheless, we could not exclude the possibility that promotion of both iTreg differentiation and nTreg expansion collaboratively contributed to the increased number of Tregs in BLM-treated mice.

Although BLM enhanced the number of Tregs, we were not able to detect any alteration in the proportion of CD4^+^ T cells, CD8^+^ T cells, and Gr1^+^CD11b^+^ myeloid-derived suppressor cells (MDSCs) in the spleen of BLM-treated mice (data not shown). This is consistent with a previous report^9^. It was also reported that BLM increased the number of INFγ^+^CD8^+^ CTLs in a mouse tumor model^9^. We confirmed this observation and found that BLM treatment enhanced the number of tumor-infiltrating IFNγ-expressing CD8^+^ T cells in B16-F10 melanoma-bearing mice (data not shown). However, our results did not show a further enhanced expansion of INFγ^+^ CTLs after CY treatment (data not shown). Presumably, BLM treatment at the dose used in this study achieved a maximal expansion of INFγ^+^ CTLs and thus any additional effect of CY could not be detected.

Recent studies have shown that some chemotherapeutic agents eliminated Tregs in cancer patients. For example, docetaxel and gemcitabine can deplete circulating Tregs^30^. Among these Treg-eliminating chemotherapeutic agents, the effect of CY has been well-studied, providing compelling evidence that it eliminated tumor-associated TNFR2-expressing Tregs in both human patients and in mouse tumor models^15^. In this study, the combination of BLM and CY showed a synergetic anti-tumor effect on mouse B16-F10 melanomas. Although this effect was likely attributable to the reduction in Tregs, other actions of these 2 chemotherapeutic agents cannot be excluded. For example, it is known that BLM causes DNA breaks similar to the effect of radiotherapy^31^. Active metabolites of CY can cause DNA cross-linking and thus result in cell death^16^. Furthermore, both BLM and CY have been shown to induce immunogenic cell death^32^. The contribution of these effects of BLM and CY to the observed synergistic anti-tumor action should be addressed in future studies.

## Conclusions

Taken together, our study showed that CY completely abrogated the expansion of Tregs induced by BLM. This effect may be, at least partially, responsible for the synergistic effect of BLM and CY in the treatment of mouse B16-F10 melanomas. Our results suggested that these 2 chemotherapeutic agents may represent a safer and more effective combination therapy for the treatment of cancer patients, and thus merit future study.
