# Supplementary material for: Cyclophosphamide abrogates the expansion of CD4+Foxp3+ regulatory T cells and enhances the efficacy of bleomycin in the treatment of mouse B16-F10 melanomas
Source: Cancer Biol Med. 2021 Aug 11;18(4):1010–20. doi: 10.20892/j.issn.2095-3941.2021.0027 (PMC8610150; doi:10.20892/j.issn.2095-3941.2021.0027)
Supplement: Supplementary file 1 [file cbm-18-1010-s001.pdf]

Supplementary materials

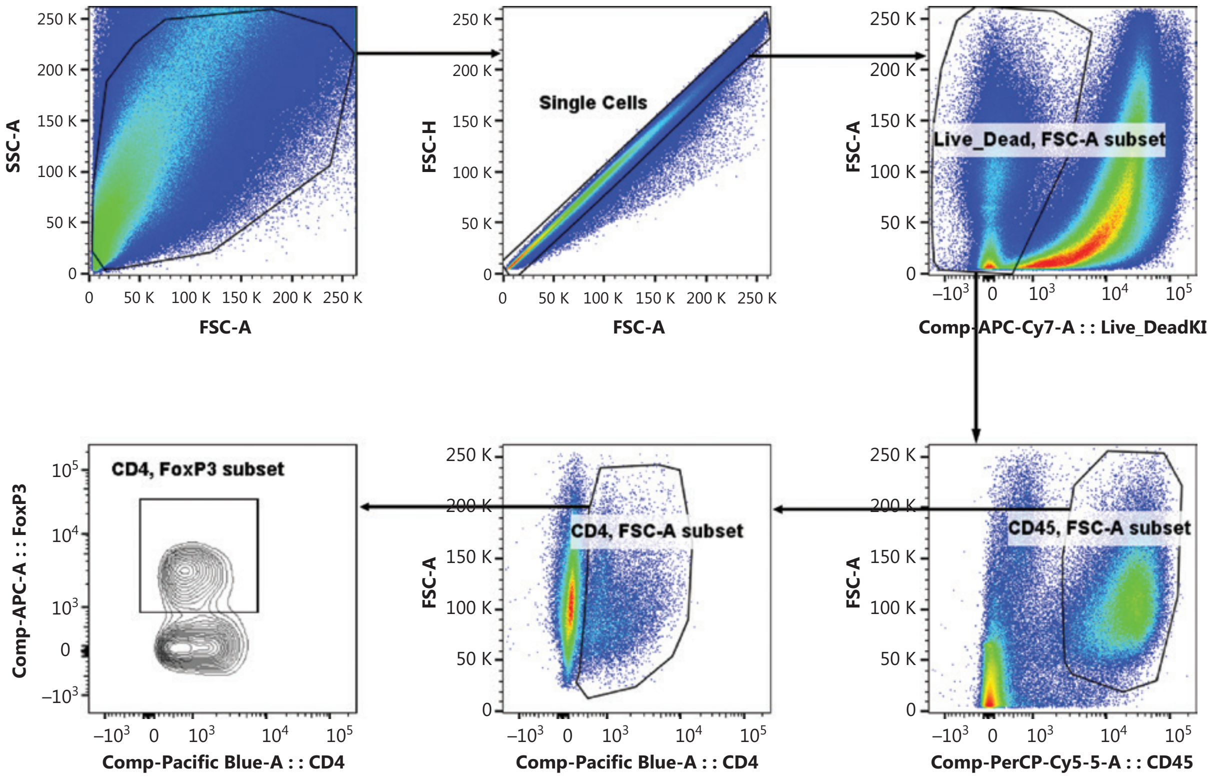

Figure S1 Gating strategy for fluorescence-activated cell sorting of tumor infiltrating regulatory T cells ($CD4^+FoxP3^+$).

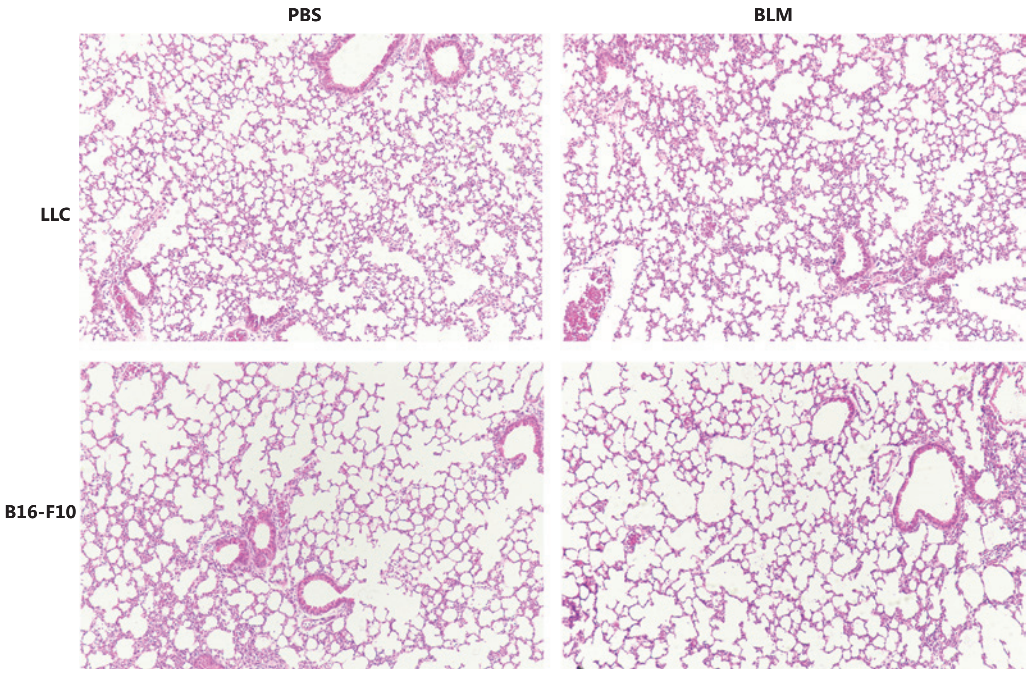

Figure S2 Effects of a low dose of bleomycin on lungs of LLC and B16-F10 tumor-bearing mice. Lung tissue sections were stained with hematoxylin and eosin for pathological observation (×100).

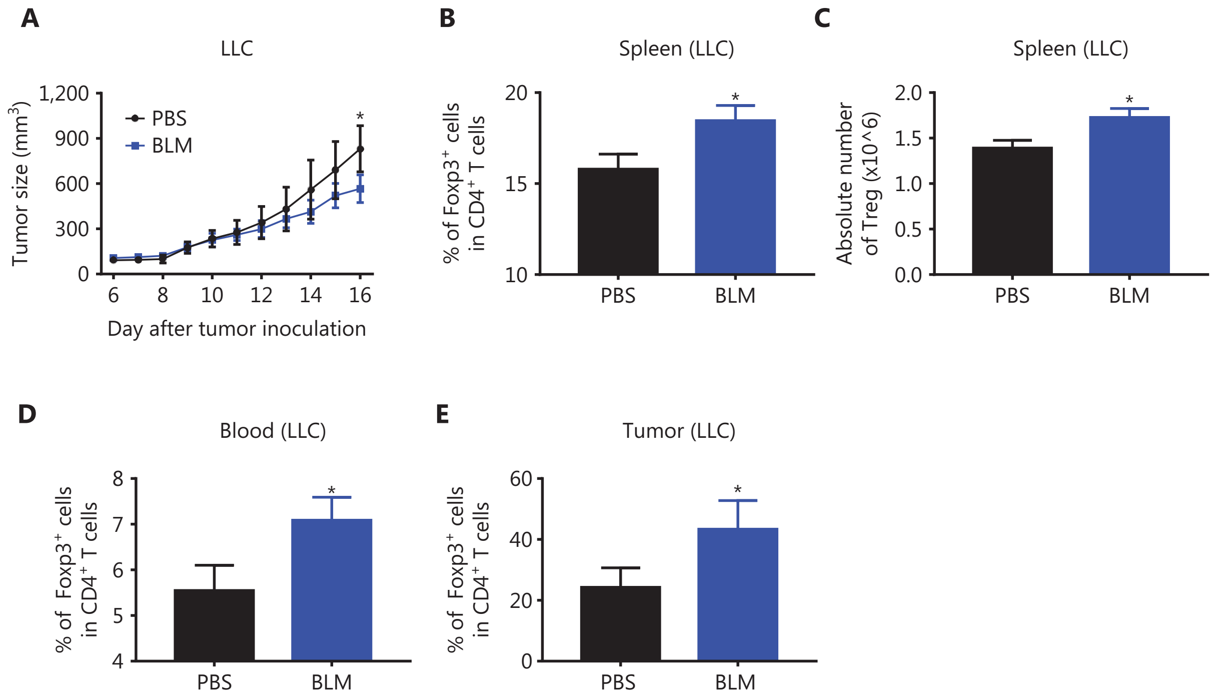

Figure S3 The effect of low dose bleomycin (BLM) on regulatory T cells (Tregs) in LLC tumor-bearing mice. C57 BL/6 mice were inoculated in the right flank with LLC tumor cells [5 × 10^5 cells in 0.1 mL of phosphate-buffered saline (PBS)]. On day 6 after tumor inoculation, mice were treated with PBS, or 2 mg/kg BLM every 3 days for a total of 4 doses. On day 16 after tumor inoculation, the percentages of Tregs in $CD4^+$ T cells present in the spleen, blood, and tumor were analyzed by fluorescence-activated cell sorting. (A) The growth curves of LLC tumors in mice. (B–E) The percentage of Tregs in $CD4^+$ T cells present in the spleen (B), blood (D), tumor tissue (E), and the absolute number of Tregs in the spleen (C) in the LLC tumor model. Data (means ± SD, N = 5) shown are representatives of 2 separate experiments. *P < 0.05 *vs*. the PBS treatment group.

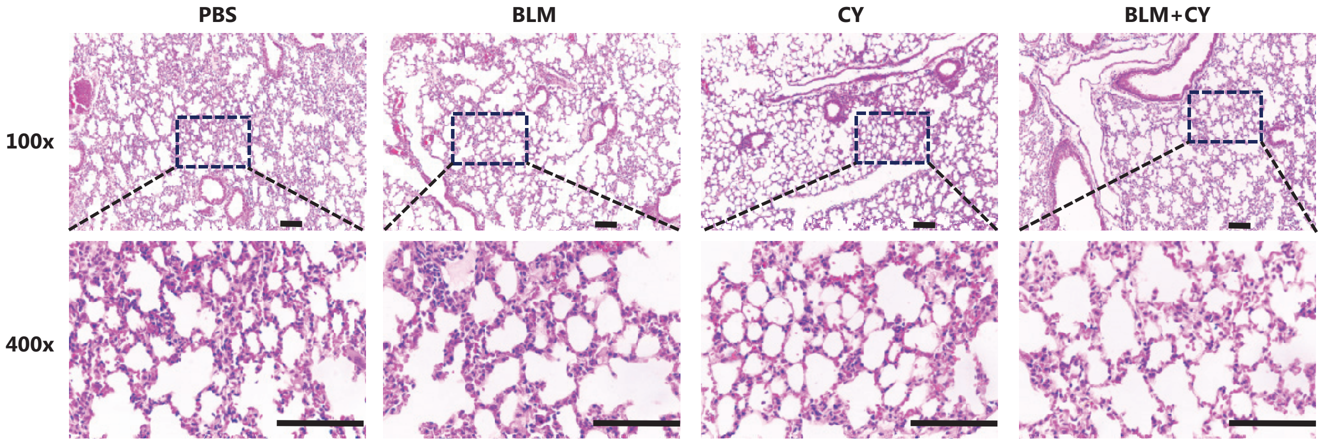

Figure S4 The effects of low dose of bleomycin, cyclophosphamide, and combination therapy on the lungs of B16-F10 melanoma-bearing mice. Lung tissue sections were stained with hematoxylin and eosin for pathological observation (×100 and ×400). The scale bar indicates 10 μm.
